# Editorial

**Published:** 2016-02-02

**Authors:** Shibal Bhartiya

‘Science is simply common sense at its best, that is, rigidly accurate in observation, and merciless to fallacy in logic.’

— Thomas Huxley

The Editorial Team of Journal of Current Glaucoma Practice (JOCGP) takes great pride in bringing to you the current issue and hope that you will find it both insightful and relevant to your clinical practice.

Faiq et al reviewed the current understanding of how mutations in CYP1B1 gene lead to disease phenotype in primary congenital glaucoma (PCG). They are of the view that the currently available functional characterization and molecular modelling studies may help in understanding the CYP1B1-mediated pathogenesis and, over time, may help in devising mechanisms to curb primary congenital glaucoma. It is also relevant to note that expressing the gene and its relevant mutants in heterologous hosts and subjecting to various tests is likely to yield important information that may prove essential in development of novel treatments for glaucoma in children and neonates.

In a related review, Chan et al provide a broad overview on the pathophysiology and diagnostic approach to primary congenital glaucoma with major emphasis on the treatment options of PCG. While reviewing the well-established treatment options, namely goniotomy, trabeculotomy and combined trabeculotomy-trabeculectomy, they also discuss the recent updates on secondary treatments: trabeculectomy, antimetabolites, glaucoma-drainage devices and cyclodestructive procedures. The review aims to discuss the diagnostic and therapeutic challenges, and provides an update on PCG management with current evidence.

The role of diabetes mellitus (DM) in glaucoma etiology or progression remains inconclusive, as reviewed by Costa et al. The authors also highlight the fact that this remains so despite the strong correlation in the experimental models based on animal-induced chronic hyperglycemia which can be explained by common neurodegenerative mechanisms. They also discuss the need to further study the effect of diabetes on wound healing in glaucoma, with respect to outcomes of more recent surgical procedures and the almost mandatory use of antifibrotics.

The functional evaluation of glaucoma patients by analyzing the visual field is essential to determine severity and progression of the disease, and the visual field index (VFI) had a strong correlation with mean deviation (MD); however, this correlation was weaker in mild disease, as some patients with early disease had very high VFI values (ceiling effect). Therefore, Sousa et al report that initial deterioration in visual field status (as assessed by MD values) in patients with early disease may not be detectable using the index alone.

Rodriguez et al determine the progression of pigment dispersion syndrome into pigmentary glaucoma in a Colombian population rate of progression is similar to that reported for other ethnic groups. They also report that an intraocular pressure (IOP) > 21 mm Hg was the only risk factor associated with progression in this sample.

Bikbov et al evaluate the techniques and clinical outcomes of Ahmed Glaucoma Valve implantation, especially with adjunct antimetabolite and anti-VEGF drugs, in cases of refractory glaucoma. They also discuss the experimental and histological examinations of the filtering bleb encapsulation in this context, and suggest methods for refining the operative technique which may help in better outcomes.

In a related article, Groth et al assess the utility of viscoelastic injection to induce bleb expansion and decrease IOP in eyes with encapsulated glaucoma tube shunt blebs and report that the procedure may be used to successfully restore encapsulated bleb function, providing a substantial (~10 mm Hg) IOP decrease into the mid-normal pressure range. This approach may, therefore, be considered as a surgical option to facilitate long-term pressure control in patients with intractable ocular hypertension after tube shunt surgery.

In a case series of three patients with previous trabeculectomies who developed elevated IOP in the immediate postoperative period after routine Descemet stripping automated endothelial keratoplasty (DSAEK), Sheales et al find that the mechanism for this pressure rise is uncertain but may involve air in the trabeculectomy’s sclerostomy or bleb resulting in blockage of aqueous flow.

As always, we look forward to hearing from you.

**Shibal Bhartiya**

